# Silent Storm Unveiled: Lupus Nephritis and Cerebral Vasculitis in Systemic Lupus Erythematosus

**DOI:** 10.7759/cureus.57751

**Published:** 2024-04-07

**Authors:** Rucha Sawant, Shilpa A Gaidhane, Vrushali Mahajan, Pranav Chaudhari, Gautam N Bedi

**Affiliations:** 1 Internal Medicine, Jawaharlal Nehru Medical College, Datta Meghe Institute of Higher Education & Research, Wardha, IND; 2 School of Epidemiology and Public Health, Jawaharlal Nehru Medical College, Datta Meghe Institute of Higher Education & Research, Wardha, IND; 3 Pathology, Alexis Multispecialty Hospital, Nagpur, IND

**Keywords:** lupus nephritis, neuropsychiatric sle, anti-double-stranded dna, autoimmunity, sle

## Abstract

Systemic lupus erythematosus (SLE) is a chronic autoimmune disorder known for its intricate clinical manifestations, spanning a spectrum of symptoms, including neuropsychiatric SLE (NPSLE) and lupus nephritis (LN). This condition predominantly affects young women of childbearing age, presenting a diverse array of symptoms that pose challenges in both diagnosis and treatment. Diagnosing central nervous system (CNS) involvement in SLE remains notably difficult despite being rooted in an autoimmune inflammatory response driven by cytokine surges. There exists no single definitive test for diagnosis, necessitating a thorough evaluation of clinical presentations, neurological indicators, and specific antibody detection. LN typically evades diagnosis until the patient progresses to a state of advanced renal insufficiency, demanding aggressive therapeutic interventions. In this discourse, we examine a case marked by generalized tonic-clonic seizures. While epilepsy might be initially suspected, in this instance, the underlying cause lay deeper, concealed within the complexities of autoimmune dysregulation. Additional symptoms included generalized edema, sun-exposed rash, oral ulcers, and recurrent fever over the past six months. The puzzle pieces eventually coalesced through meticulous examination of each clinical manifestation, coupled with laboratory analyses, neuroimaging studies, and renal biopsy, revealing a complex scenario of cerebral vasculitis concurrent with LN in a case of SLE.

## Introduction

Patients diagnosed with systemic lupus erythematosus (SLE) exhibit elevated levels of autoantibodies and immune complexes in their bloodstream, posing a potential threat to any organ. The precise etiology of SLE remains elusive, yet it encompasses a complex interplay of genetic predisposition, environmental influences, and hormonal factors. Furthermore, a positive family history and other autoimmune predispositions contribute to the risk profile.

Neuropsychiatric systemic lupus erythematosus (NPSLE) represents a subtype of SLE characterized by both neurological and psychiatric manifestations. While not exhaustive, NPSLE often arises from various vascular abnormalities [1]. These anomalies span from abnormalities in small blood vessels leading to microinfarctions and microhemorrhages to instances of thromboembolism [1]. True vasculitis, although less frequent, may affect larger vessels. Given the clinical heterogeneity of NPSLE, observed in approximately 10-80% of patients, identifying its causative factors proves challenging, compounded by a dearth of evidence-based treatments, thereby complicating overall disease management [1].

Conversely, lupus nephritis (LN), a consequence of immune complex deposition within the glomeruli, often presents with subtle signs and is frequently overlooked unless specifically investigated [2]. Consequently, screening SLE patients for LN during the initial diagnostic workup is imperative. This evaluation typically entails urine analysis, kidney function assessment, and glomerular filtration rate (GFR) estimation. Renal biopsy becomes warranted when proteinuria exceeds 500mg/dl [2]. In this context, we describe the case of a young female presenting with neurological symptoms, which, upon meticulous investigation, revealed a diagnosis of SLE accompanied by LN and NPSLE.

## Case presentation

**Figure 1:**
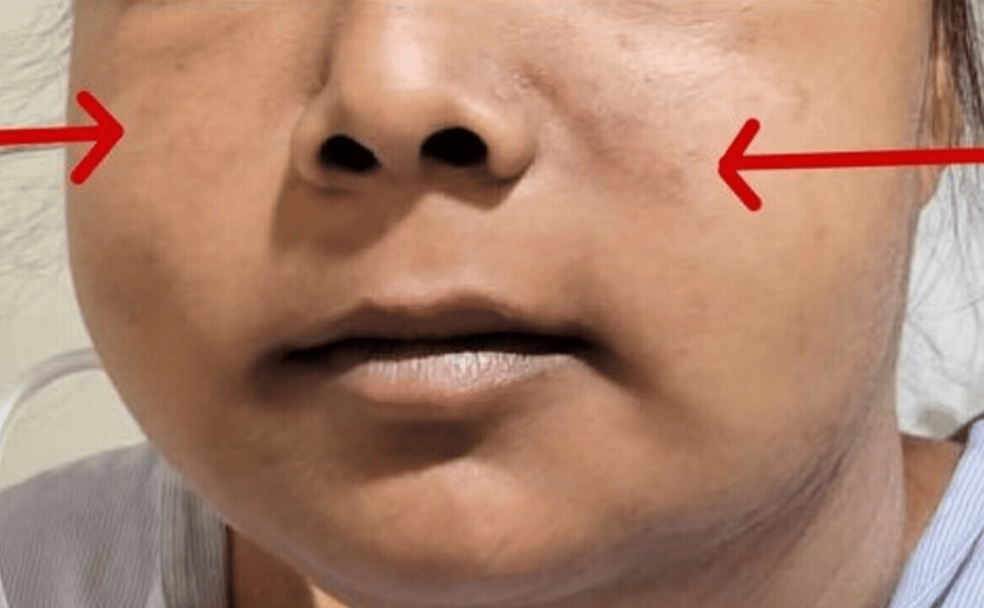
Bilateral malar rash sparing the nasolabial region.

An 18-year-old young woman presented to the emergency department experiencing a generalized tonic-clonic seizure (GTCS), which persisted for approximately four minutes. The seizure was successfully terminated using intravenous lorazepam at a dose of 0.1 mg/kg administered over a five-minute period. Information provided by her mother, acting as the primary informant, revealed that similar episodes of GTCS had occurred twice the previous day, prompting symptomatic treatment at a local healthcare facility. Upon regaining consciousness, the patient herself reported a history of fever, oral ulcers, and facial puffiness and swelling over the course of the past six months. She also complained of easy fatiguability and reduced appetite. The fever had a gradual onset and was low grade, intermittent, devoid of associated chills, and typically subsided following over-the-counter antipyretic medications. The oral ulcers were characterized by their painless nature. The swelling initially manifested in the periorbital region and progressively spread throughout her body. Following the postictal phase, the patient underwent an examination. She presented as extremely irritable and afebrile with a heart rate of 100 beats per minute, blood pressure measuring 170/100 mmHg, and a respiratory rate of 30 cycles per minute, maintaining an oxygen saturation level of 92 percent on room air. She exhibited a hyperpigmented rash on her malar area, with the nasolabial folds notably spared (Figure 1).

**Figure 2:**
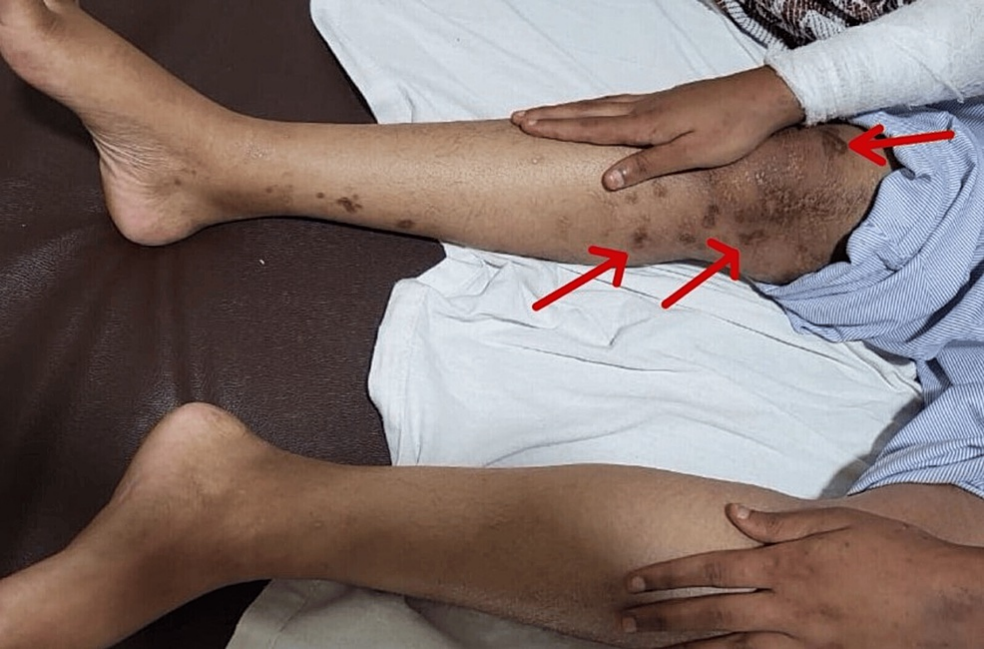
Right knee with a maculopapular erythematous rash.

She also had reduced hair density by thinning hair strands, and her right knee also had a maculopapular erythematous rash (Figure 2).

**Figure 3:**
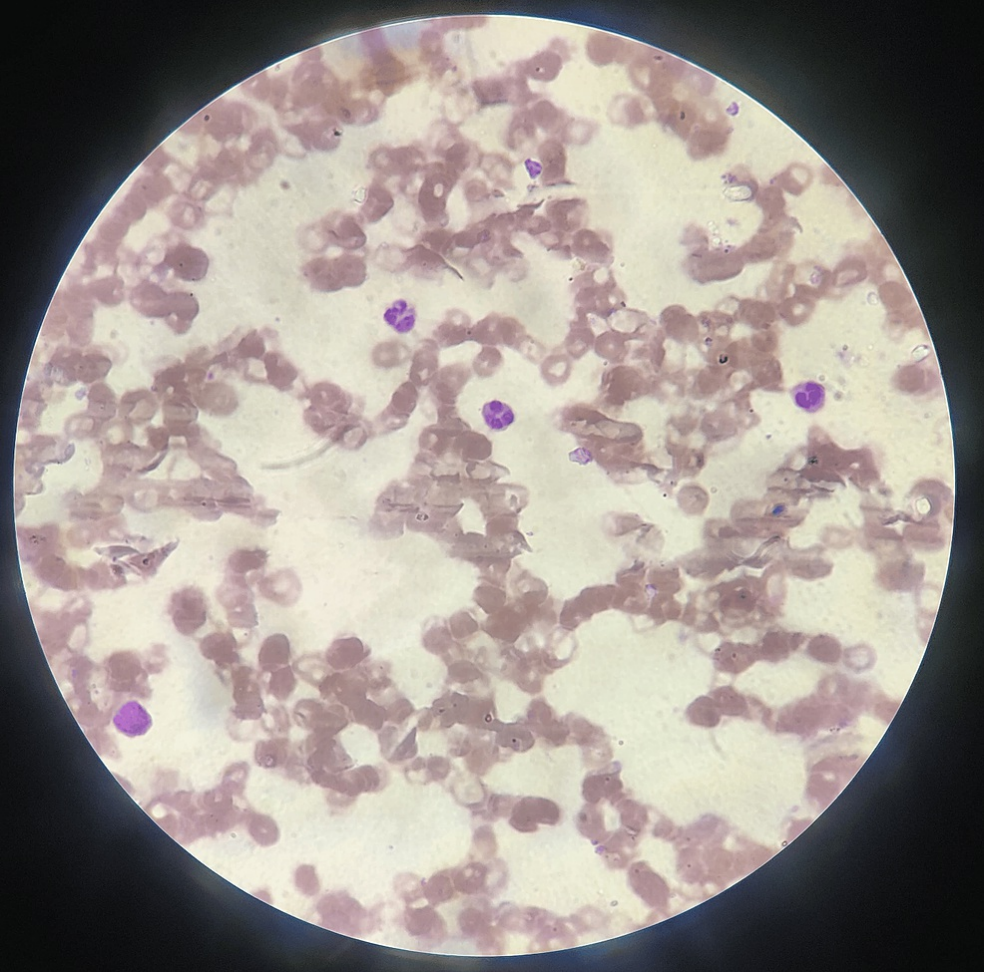
Peripheral smear shows normocytic hypochromic anemia with anisopoikilocytosis, showing tear drop cells and ovalocytes.

Upon auscultation, diminished air entry was noted in the intrascapular, axillary, and inframammary regions. Abdominal examination revealed abdominal distension accompanied by the presence of a fluid thrill. Evaluation of the neurological and cardiovascular systems yielded normal findings. Routine blood analysis revealed evidence of anemia and leukocytopenia. Peripheral smear examination displayed features consistent with normocytic hypochromic anemia characterized by anisopoikilocytosis, including tear drop cells and ovalocytes (Figure 3).

Furthermore, laboratory investigations revealed elevated levels of creatinine and urea, accompanied by increased serum concentrations of potassium, uric acid, phosphate, total bilirubin, and indirect bilirubin. Conversely, there was a reduction in serum albumin, calcium, and vitamin D levels. Analysis of a spot urine protein-to-creatinine ratio (UPCR) demonstrated proteinuria measuring 200.5 mg/mmol. Subsequent testing revealed significantly elevated erythrocyte sedimentation rate (ESR) and C-reactive protein (CRP) levels. Immunofluorescence testing for antinuclear antibody (ANA) yielded positive results, with a titre of 1:640 displaying a cytoplasmic pattern. In addition, anti-double-stranded DNA (ds-DNA) testing returned positive. The Coombs test was positive, and serum lactate dehydrogenase (LDH) levels were elevated. ANA by immunofluorescence also indicated low complement levels of complement 3 (C3) and complement 4 (C4). Based on these comprehensive findings, a diagnosis of SLE was confirmed following the criteria established by the European League Against Rheumatism (EULAR) and the American College of Rheumatology (ACR). Notably, tests for anticardiolipin antibodies IgG and anti-β2-glycoprotein-1 (β2GPI) antibodies returned negative results. Tests for HBsAg and anti-HCV and ANCA antibodies were negative. The UPCR was also elevated (Table 1).

**Table 1:** Laboratory findings MCV: mean corpuscular volume, WBC: white blood cell, INR: international normalized ratio, AST: aspartate transaminase, ALT: alanine transaminase, ANA: antinuclear antibody, ds-DNA: double-stranded DNA, C3: complement 3, C4: complement 4, ESR: erythrocyte sedimentation rate, CRP: C-reactive protein, LDH: lactate dehydrogenase, eGFR: estimated glomerular filtration rate

Laboratory parameters	Patient values - on admission	Patient values - on discharge	Patient values - on follow-up	Reference range
Hemoglobin (g/dl)	7.2	9.1	10.1	12-18
MCV	72	84	82	81-102
WBC (/mm^3^)	3600	4600	5200	4500-11,000
Total platelet (x 10^9^/L)	1.67	2.2	2.7	1.4-4.5
INR	1.1	1.0	1.0	1.01
Urea (mg/dl)	231	65	42	9-22
Creatinine (mg/dl)	3.8	2.8	2.0	0.6-1.25
Sodium (mg/dL)	142	144	146	137-144
Potassium (mg/dL)	4.3	4.5	4.6	3.5-5.2
Magnesium (mg/dL)	1.7	1.8	1.8	1.7-2.2
Phosphate (mg/dL)	5.1	4.9	4.6	2.5-4.4
Calcium (mg/dL),	7.1	7.4	7.8	8.4-10.1
Total bilirubin (mg/dL)	1.8	0.4	0.4	0.2-1.3
Direct bilirubin (mg/dL)	0.2	0.2	0.2	0-0.3
(AST) (U/L)	39	35	38	<50
(ALT) (U/L)	40	44	46	17-59
24-hour urinary protein (mg/day)	650	480	-	24-240
Urinary protein to creatinine ratio (mg/g)	200.5	160	142	< 150
Total protein (g/dL)	3.1	4.2	5.3	6 to 8.3
Serum albumin (g/dL)	1.6	2.2	2.9	3.4 to 5.4
Serum ferritin (ng/ml)	17	-	-	17.5-464
Serum iron (mcg/dl)	38	-	-	49-181
Direct total iron binding capacity (mcg/dl)	500	-	-	261-462
Vitamin B12 (pg/ml)	150	700	-	239-913
ANA	Positive titre -1:640	-	-	< 1:160
ds-DNA (IU/dl)	2.1	-	-	<0.9
C3 (mg/dl)	26	86	112	80-165
C4 (mg/dl)	8	12	36	14-44
ESR by winterobe method (mm/hr)	72	25	19	0-20 mm
CRP (mg/dl)	154	30	1.1	<1
Serum LDH (U/L)	2022	745	330	120-246
Direct coombs test	Positive	-	-	
eGFR (/ml/min/1.73 m^2^)	17	24	36	>90

**Figure 4:**
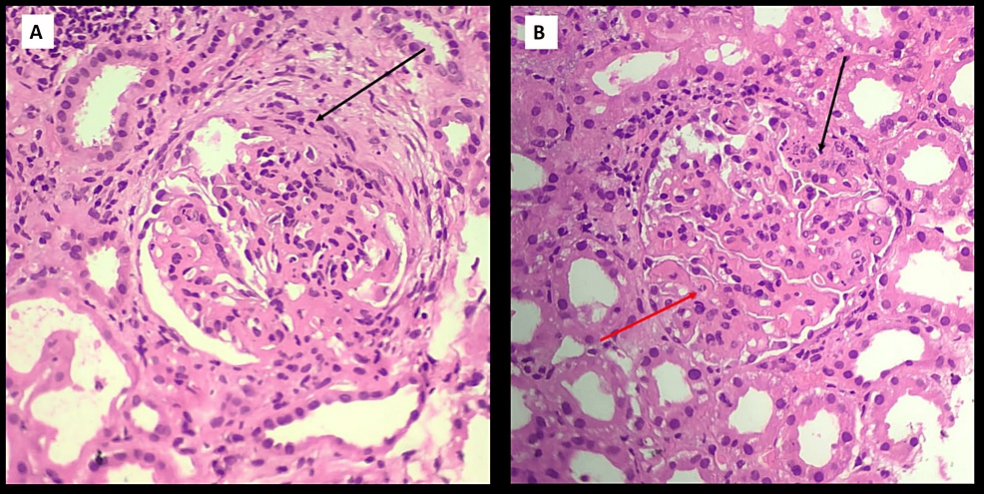
A: Microphotograph showing a hypercellular glomerulus with mesangial and endocapillary hypercellularity with occasional neutrophils (H&E 40X). The glomerular basement membrane is irregularly thickened. The arrow points to a segmental fibrocellular crescent. B: Microphotograph showing a glomerulus that appears hypercellular with lobular accentuation and endocapillary hypercellularity (H&E 40X). Many neutrophils/karyorrhectic debris can be appreciated at the black arrow. The red arrow points to a wire loop lesion. Surrounding tubules show injury with mixed inflammatory infiltrate in the interstitium.

**Figure 5:**
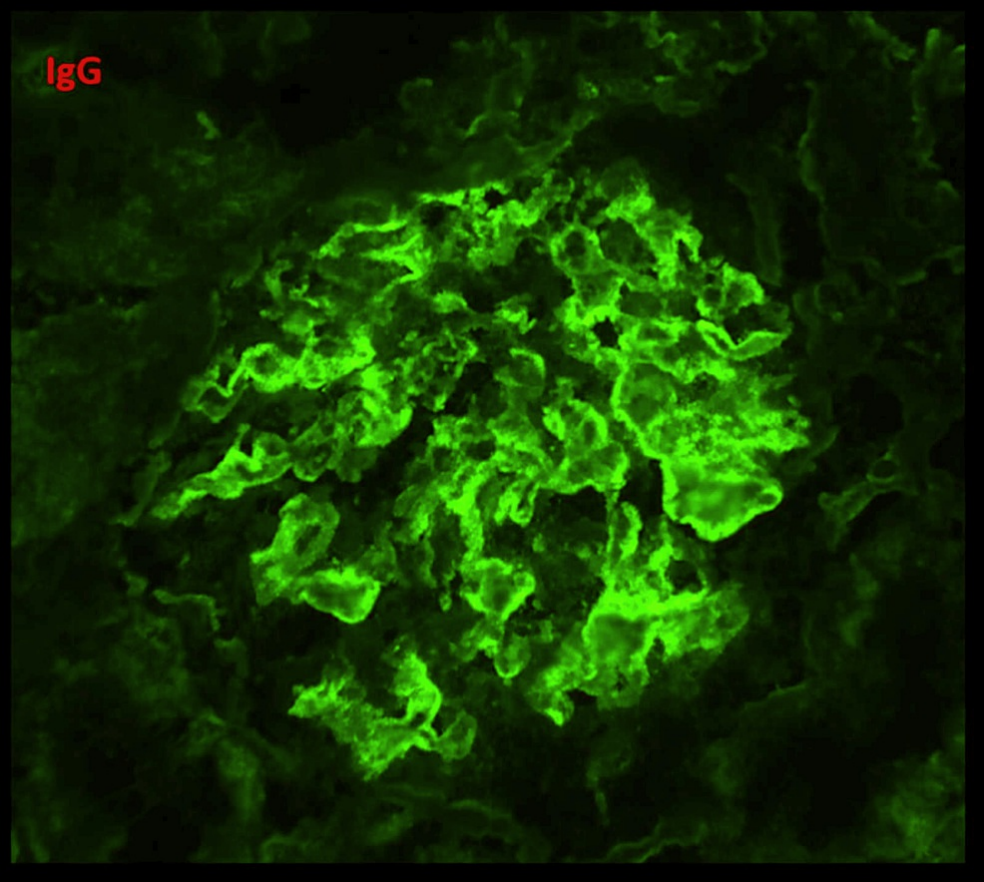
Immunofluorescence microscopy (IgG, 40X). The IF image is showing a glomerulus with diffuse fine granular deposits of 2+ to 3+ intensity along the GBM, indicative of subepithelial immune deposits. These deposits were seen over >50% of the total GBM area. Few segments show linear interrupted/semi-linear deposits along the GBM indicative of subendothelial immune deposits. Segmental mesangial deposits were also present. IgG: immunoglobulin G, GBM: glomerular basement membrane

**Figure 6:**
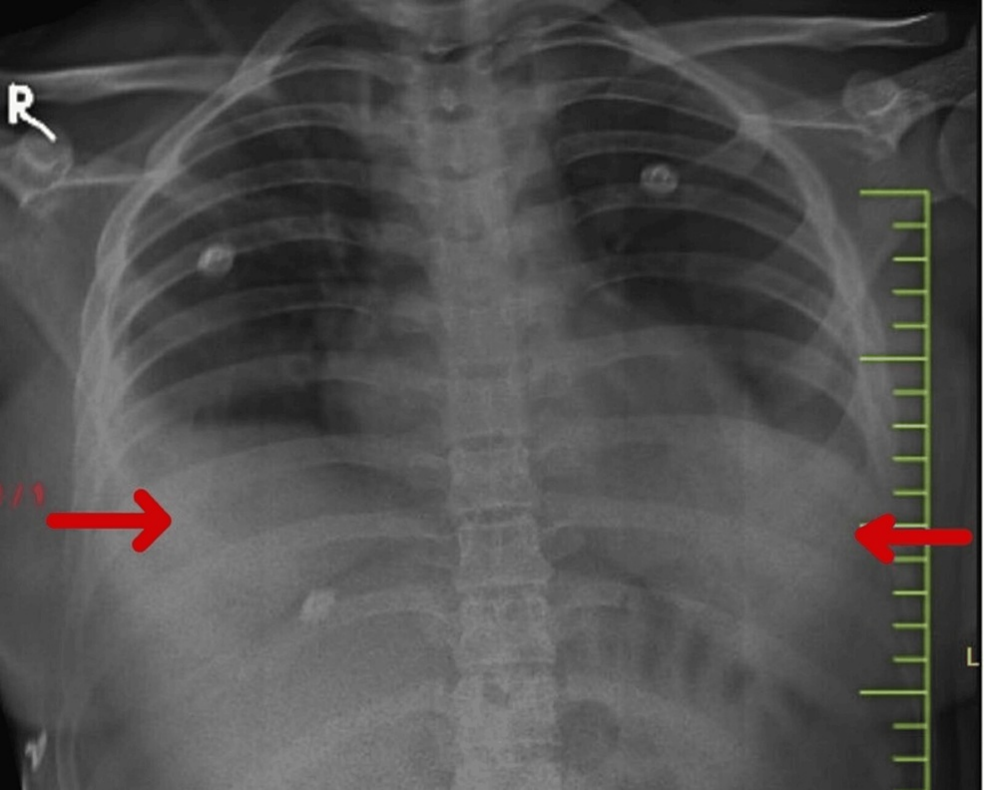
Chest X-ray showing blunting of the bilateral costophrenic angle.

With the 24-hour urinary protein level elevated at 650 mg/day, suspicion of LN arose, prompting a renal biopsy for further evaluation. The biopsy results revealed a combination of class IV diffuse proliferative glomerulonephritis and class V membranous nephropathy, thereby confirming the diagnosis of LN (Figures 4, 5). A chest X-ray was done, which suggested bilateral pleural effusion (Figure 6). Her sputum examination was done, cultures showed no growth or organism, and her GeneXpert did not detect mycobacterium tuberculosis.

Abdominal ultrasonography revealed bilateral kidneys with heightened cortical echotexture, indicative of renal involvement. In addition, splenomegaly measuring 16 cm and gross ascites were noted. Fifty mL of clear ascitic fluid was aspirated and sent for investigation. Ascitic fluid analysis showed no red blood cells or malignant cells and 55 cells/cumm. Adenosine deaminase was 3.5 U/L (8.6-20.5 U/L). Serum ascites albumin gradient (SAAG) was more than 1.1. Cultures were found to have no growth of microorganisms. Ascitic fluid pH was 7.4, albumin was 0.7 g/dL, LDH was 250 U/l, and sugars were 100 mg.

**Figure 7:**
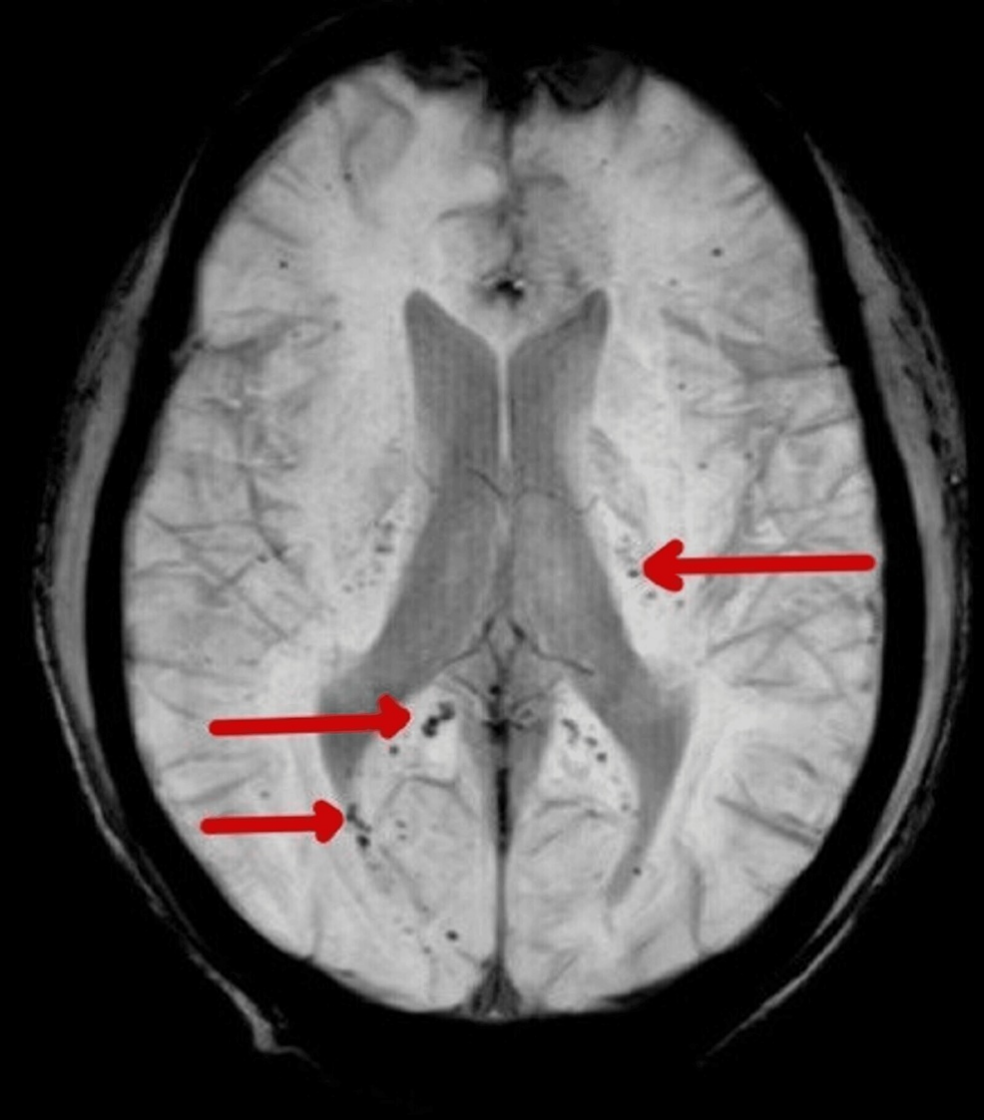
Susceptibility-weighted MRI shows multiple petechial hemorrhages involving bilateral halves of the splenium of the corpus callosum and bilateral internal capsule.

The patient remained extremely irritable during the hospital stay and had difficulty falling asleep. Subsequent MRI imaging of the brain exhibited multiple microhemorrhages alongside hyperintensities consistent with cerebral vasculitis changes (Figure 7).

The patient's treatment regimen commenced with oral administration of hydroxychloroquine (HCQ) at a dose of 100 mg once daily, alongside intravenous methylprednisolone (MP) administered at 500 mg once daily for a duration of three days, followed by a transition to oral prednisolone at 40 mg once daily. She was given an Injection of mesna 200 mg in 50 ml of 0.9% normal saline over 30 minutes; after one hour, she was given an injection of cyclophosphamide 500 mg in 400 ml of 0.9% normal saline over four hours. This was repeated every 14 days for a total of six doses. Her blood pressure was controlled with tablet nifedipine 10mg thrice a day. The patient received one unit of packed red blood cells and was prescribed sevelamer tablets at 400 mg twice daily. No calcium correction was deemed necessary given the corrected calcium level for albumin at 9 mg/dl. To manage seizures, the patient was prescribed levetiracetam tablets at 500 mg twice daily. During the hospital stay, the patient did not experience any additional seizures. Furthermore, in view of anemia, parenteral iron sucrose was administered at a dose of 100 mg daily for a period of six days, while intramuscular vitamin B12 was given at a dose of 1000 µg once daily for seven days. In addition, the patient received 200 ml of injectable 20% albumin infused at a rate of 20 ml/hr.

After a period of seven days in the hospital, she improved in such a way that her fatigue was reduced, and her appetite improved. Her vitals were stabilized, and she was subsequently discharged. She was advised to follow up in a week.

The patient followed up after two months. Upon follow-up, she had significantly reduced edema. Her mother told history that she regained her appetite, was not irritable, and slept properly. She had even resumed her education. Her vitals were found to be within normal limits. Her laboratory parameters have improved since then. She was advised to follow up after 14 days.

## Discussion

SLE is a complex autoimmune disorder characterized by diverse clinical and laboratory manifestations, a variable disease course, and an unclear underlying cause [3]. Constitutional symptoms commonly associated with SLE encompass myalgia, fluctuations in weight, and fever. While SLE patients frequently report fever, distinguishing disease-related activity from other etiologies, such as infections, can present challenges. A diminished leukocyte count during fever episodes may suggest lupus activity. Mucocutaneous manifestations often include the classic "butterfly rash," erythematous, affecting the malar region but sparing the nasolabial folds, typically exacerbated by sun exposure. In addition, manifestations may involve discoid rash, which is prone to scarring, non-scarring alopecia, photosensitivity, and oral or nasal ulcers. Vasculitis prevalence among SLE patients ranges from 11% to 36%, with small vessel vasculitis being the most prevalent, presenting as palpable purpura, petechiae, and nodular lesions [4].

NPSLE encompasses a broad spectrum of features ranging from strokes and seizures to psychosis and peripheral neuropathies. The incidence of NPSLE in SLE patients varies between 37% and 90%. Within CNS involvement, cognitive impairments account for 55-80%, headaches for 24-72%, and cerebral vascular events for a minority, ranging from 5% to 18% [4]. Non-inflammatory forms of CNS vasculopathy, primarily driven by autoantibodies like anticardiolipin, anti-P-ribosomal, and anti-NMDA antibodies, are predominant. Inflammatory vasculopathy affecting microvasculature is less common, with microinfarcts and thrombi more prevalent in antiphospholipid antibody-positive patients. Vasculitis of cerebral vessels occurs in less than 10% of SLE cases [5]. The patient under discussion exhibits both microvascular involvement in the form of microhemorrhages and cerebral vasculitis.

LN is a common manifestation of immune complex-mediated glomerulonephritis. Pathogenesis involves heightened interferon expression, myeloid cell activation, and neutrophil involvement [2]. Patients with LN often exhibit impaired neutrophil extracellular trap (NET) degradation [6]. Complement activation plays a significant role in endothelial damage, amplifying inflammation [7]. Deposition of various components, including C1q, Sm, La (SS-B), Ro (SS-A), ribosomes, and ubiquitin [8]. The immune complexes can be deposited in the mesangium and subendothelial or subepithelial areas [8,9]. Genetic associations, particularly with HLA-DR3 and DR15, increase susceptibility to LN in SLE patients [10]. Roughly half of SLE patients develop LN, with approximately 10% progressing to end-stage renal disease (ESRD) [2]. Renal abnormalities typically emerge within six months to three years, often presenting as nephrotic range proteinuria and elevated plasma creatinine levels [2]. Renal biopsy is warranted in suspected LN cases to guide histopathological grading and exclude alternative pathologies.

Hematological abnormalities in SLE typically affect all three blood cell lines. While autoimmune hemolytic anemia is uncommon in SLE patients, the most prevalent type of anemia observed is anemia of chronic illness [11]. Leukopenia, resulting from either lymphopenia or secondary neutropenia, is present in up to half of SLE patients and is a direct indicator of disease activity [12]. Mild thrombocytopenia, when encountered, rarely necessitates intervention. Lymphadenopathy and splenomegaly may occur, particularly during disease flare-ups. Thromboembolic events are increasingly associated with patients harboring antiphospholipid antibodies and lupus anticoagulants, manifesting as either venous or arterial thrombosis, with the latter being relatively more common [13].

Arthritis and arthralgias, characterized by painful, symmetrical, migratory joint involvement affecting multiple sites, are among the earliest and most prevalent features observed in over 90% of SLE patients [14]. Cardiac manifestations include pericarditis, Libman-Sacks endocarditis involving the valves, coronary artery disease, and occasionally myocarditis. Raynaud phenomenon may be present in up to half of SLE patients [15]. Ophthalmic manifestations, primarily keratoconjunctivitis sicca secondary to Sjogren's syndrome, followed by lupus vasculopathy, are common [16]. Gastrointestinal symptoms, encompassing lupus hepatitis, mesenteric vasculitis, protein-losing enteropathy, and pancreatitis, are not uncommon. Pulmonary involvement may manifest as pleuritis, pulmonary hypertension, interstitial lung disease, and occasionally shrinking lung syndrome, with diffuse alveolar hemorrhage posing the gravest threat.

Once the diagnosis of SLE is established, the treatment includes hydroxychloroquine 5 mg/kg once daily and pulses of intravenous methylprednisolone. The initiation of immunosuppressive (IS) agents allows early tapering of corticosteroids and prevents further flares. Non-corticosteroid ISs are used to reduce the flare of SLE. They are used for induction and maintenance therapy. The most commonly used are methotrexate (MTX) and azathioprine (AZA) and mycophenolate mofetil (MMF). Fatal organ and system involvement, such as in LN and NPSLE, necessitates more intensive parenteral therapy with CYC or MMF in conjunction with corticosteroids [17]. Belimumab and anifrolumab are biologics that have been licensed for the treatment of SLE [17]. Rituximab efficiently produces remission of LN in individuals who have not responded to conventional treatments [17].

## Conclusions

Neuropsychiatric complications are a frequent occurrence in individuals with SLE, although they often take a backseat to the relatively less common cerebrovascular events. In the context of SLE, prompt identification of LN holds paramount importance, as it can deter the progression to ESRD. This case report illuminates an exceedingly rare convergence: the coexistence of LN alongside cerebral vasculitis in an SLE patient. The patient initially presented with neuropsychiatric symptoms, ultimately leading to the dual diagnosis of SLE and LN. This case underscores the necessity for clinicians to entertain the possibility of SLE with neuropsychiatric manifestations and concurrent LN, particularly when faced with similar clinical scenarios in young female patients.

## References

[1] Cohen D, Rijnink EC, Nabuurs RJ (2017). Brain histopathology in patients with systemic lupus erythematosus: identification of lesions associated with clinical neuropsychiatric lupus syndromes and the role of complement. Rheumatology (Oxford).

[2] Almaani S, Meara A, Rovin BH (2017). Update on lupus nephritis. Clin J Am Soc Nephrol.

[3] Talwar D, Kumar S, Acharya S (2022). Fatal recurrent angioedema with systemic section lupus erythematosus in a young female: a disturbing decline. J Clin Diagn Res.

[4] Barile-Fabris L, Hernández-Cabrera MF, Barragan-Garfias JA (2014). Vasculitis in systemic lupus erythematosus. Curr Rheumatol Rep.

[5] Goswami D, Chatterjee S, Ahmad BI, Das S (2013). Two case reports indicating the dilemma in diagnosing lupus cerebritis. J Family Med Prim Care.

[6] Reddy H, Kashiv P, Kumar S, Sejpal KN, Acharya S (2024). A rare autoimmune confluence. Cureus.

[7] Birmingham DJ, Bitter JE, Ndukwe EG (2016). Relationship of circulating anti-c3b and anti-c1q igg to lupus nephritis and its flare. Clin J Am Soc Nephrol.

[8] Akashi Y, Yoshizawa N (1995). Participation of histones and ubiquitin in lupus nephritis. Nihon Jinzo Gakkai Shi.

[9] Mannik M, Merrill CE, Stamps LD, Wener MH (2003). Multiple autoantibodies form the glomerular immune deposits in patients with systemic lupus erythematosus. J Rheumatol.

[10] Chung SA, Brown EE, Williams AH (2014). Lupus nephritis susceptibility loci in women with systemic lupus erythematosus. J Am Soc Nephrol.

[11] Dubey A, Acharya S, Shukla S (2023). Autoimmune haemolytic anaemia, acute pericarditis with cardiac tamponade as presenting manifestations in systemic lupus erythematosus- a rare case report. J Clin Diagn Res.

[12] Newman K, Owlia MB, El-Hemaidi I, Akhtari M (2013). Management of immune cytopenias in patients with systemic lupus erythematosus - old and new. Autoimmun Rev.

[13] Sarabi ZS, Chang E, Bobba R, Ibanez D, Gladman D, Urowitz M, Fortin PR (2005). Incidence rates of arterial and venous thrombosis after diagnosis of systemic lupus erythematosus. Arthritis Rheum.

[14] Greco CM, Rudy TE, Manzi S (2003). Adaptation to chronic pain in systemic lupus erythematosus: applicability of the multidimensional pain inventory. Pain Med.

[15] Cervera R, Khamashta MA, Font J (2003). Morbidity and mortality in systemic lupus erythematosus during a 10-year period: a comparison of early and late manifestations in a cohort of 1,000 patients. Medicine (Baltimore).

[16] Poshattiwar RS, Acharya S, Shukla S, Kumar S (2023). Neurological manifestations of connective tissue disorders. Cureus.

[17] Katarzyna PB, Wiktor S, Ewa D, Piotr L (2023). Current treatment of systemic lupus erythematosus: a clinician's perspective. Rheumatol Int.

